# Fabricating THz spiral zone plate by high throughput femtosecond laser air filament direct writing

**DOI:** 10.1038/s41598-020-70997-w

**Published:** 2020-08-18

**Authors:** Zhi Zhang, Zijie Dai, Yunfei Wang, Chunyue Chu, Qiang Su, Olga Kosareva, Nan Zhang, Lie Lin, Weiwei Liu

**Affiliations:** 1 Institute of Modern Optics, Nankai University, Tianjin, 300350 China; 2 International Laser Center, Lomonosov Moscow State University, Moscow, 119991 Russia

**Keywords:** Applied optics, Optical materials and structures, Optical techniques

## Abstract

The sixth-generation wireless communication will exploit the radio band with frequencies higher than 90 GHz, reaching terahertz (THz) band, to achieve huge signal bandwidths. However, the cost-effective fabrication methods of the key components in THz band, which can compromise large scale, high precision, and high efficiency, remain great challenges at present. In this work, we have developed a high throughput fabrication method based on the femtosecond laser filament direct writing. The ability of fabricating large-scale THz elements with high precision and fast speed has been demonstrated by fabricating 100 × 100 mm^2^ spiral zone plates (SZPs), which can convert the Gaussian THz beam into vortex beam. The performance of the obtained THz vortex beam is in good agreement with the theoretical predictions. The fabrication method reported here has promising applications in fabricating various kinds of THz elements on substrates with both flat and curved surfaces.

## Introduction

At present, the fifth-generation (5G) wireless facility is being constructed with dramatic speed all over the world. The study of the sixth-generation (6G) wireless communication has also attracted extensive interests, which will use the radio band above 90 GHz, reaching terahertz (THz) band, to further extend the signal bandwidths. Highly directional electromagnetic wave is the suggested candidate to provide wireless links in 6G communication networks^1,2^. Therefore, the development of the advanced beam controlling technique is under urgent demand^3,4^.

Significant improvements on beam controlling has been made in optics, which mainly relies on the skillful design of micro-/nano-structuring elements^5–7^ and the state of art fabrication technology such as electron beam lithography^8^, extreme ultraviolet lithography^9^ and ion beam etching^10^. Fortunately, these advanced optical beam controlling methods and concepts can be directly transferred to the THz band. For example, the orbital angular momentum (OAM) of the vortex optical beam provides a new informational degree of freedom due to its infinite number of eigenstates characterized by the topological charges^11^. In the communication systems, OAM can greatly increase the volume of data transferring^12,13^. OAM has also aroused considerable attentions due to its applications in optical tweezers^14,15^ and quantum entanglement^16,17^. Generally, OAM modes can be generated by using spiral phase plates^18,19^, holographic diffraction gratings^20,21^, frequency selective metasurface^22,23^, and etc. However, compared with the research of OAM in optics, the investigations in the THz band are still lag behind.

Recently, using geometric phase elements made of space-variant birefringent slabs, Minasyan et al. produced the quasi-monochromatic THz vortex beam^24^. Wu et al. utilized a THz quarter wave plate, a spiral phase plate, and Teflon axicons to generate THz vortex Bessel beam^25^. Xie et al. employed a concentric ring metal grating and photo-generated carriers to form the THz vortex beam^26^. V-shaped metasurface antennas^27^ and photo patterned birefringence liquid crystal^28^ are also proposed to generate the THz vortex beam. It is worth emphasizing that for THz wave, due to its relatively long wavelength, strong diffraction occurs when it propagates in free space, leading to the large beam diameter. Therefore, large aperture component is required in order to facilitate the beam coupling. Besides, military and deep-space communications are often based on large size phased-array antenna, which also needs large aperture device^29^. However, the current fabrication techniques are always inefficient and cannot produce elements with large apertures, which greatly restricts the THz wave’s practical applications.

In this paper, we develop a high throughput direct writing method using the femtosecond laser air filament, which can fabricate large scale THz elements with a lateral resolution of ~ 100 μm, i.e. ~ l/30 for 0.1 THz wave. In the filamentation the pulsed laser beam propagates with a constant diameter (~ 100 μm) over a distance much longer than the Rayleigh length^30,31^, making the filament direct writing be able to fabricate structures on both flat and curved surfaces. Using the fabrication method proposed here, the large-size (100 × 100 mm^2^) THz SZPs with topological charges *l* = 0, 1 and 3 are fabricated. The fabrication time for each element is ~ 4 min. The performance of the fabricated SZPs are characterized experimentally, which are in good agreement with the design schemes.

The fabrication method proposed in this paper can fabricate structures on almost all kinds of materials using a single step. It can fabricate large area structures with a maximal writing speed of 1,200 mm^2^/min. Furthermore, due to the diffraction-free property of the femtosecond laser filament, there is no rigid requirement for the focusing control. Therefore, compared with the commercial maskless laser lithography, the laser filament direct writing technique in this paper is more robust and easier to use, which is very suitable for fabricating large area THz elements.

## Methods

### Sample preparation

We used a quartz substrate of 100 × 100 × 2 mm^3^, soaked it in acetone, methanol and ultra-pure water successively. The quartz substrate was cleaned by ultrasonic washer for about 3 min, then washed with ultra-pure water and dried with nitrogen gun. The purpose of cleaning is to remove organic contaminants from the quartz surface to obtain a clean substrate. After cleaning, we used a vacuum coating machine to plate a silver film with a thickness of ~ 60 nm on the top of the quartz substrate. During the coating process, the sample is rotated by a speed of 20 r/min and an evaporation rate of 10 Å/s is achieved.

### Fabricating SZPs

**Figure 1:**
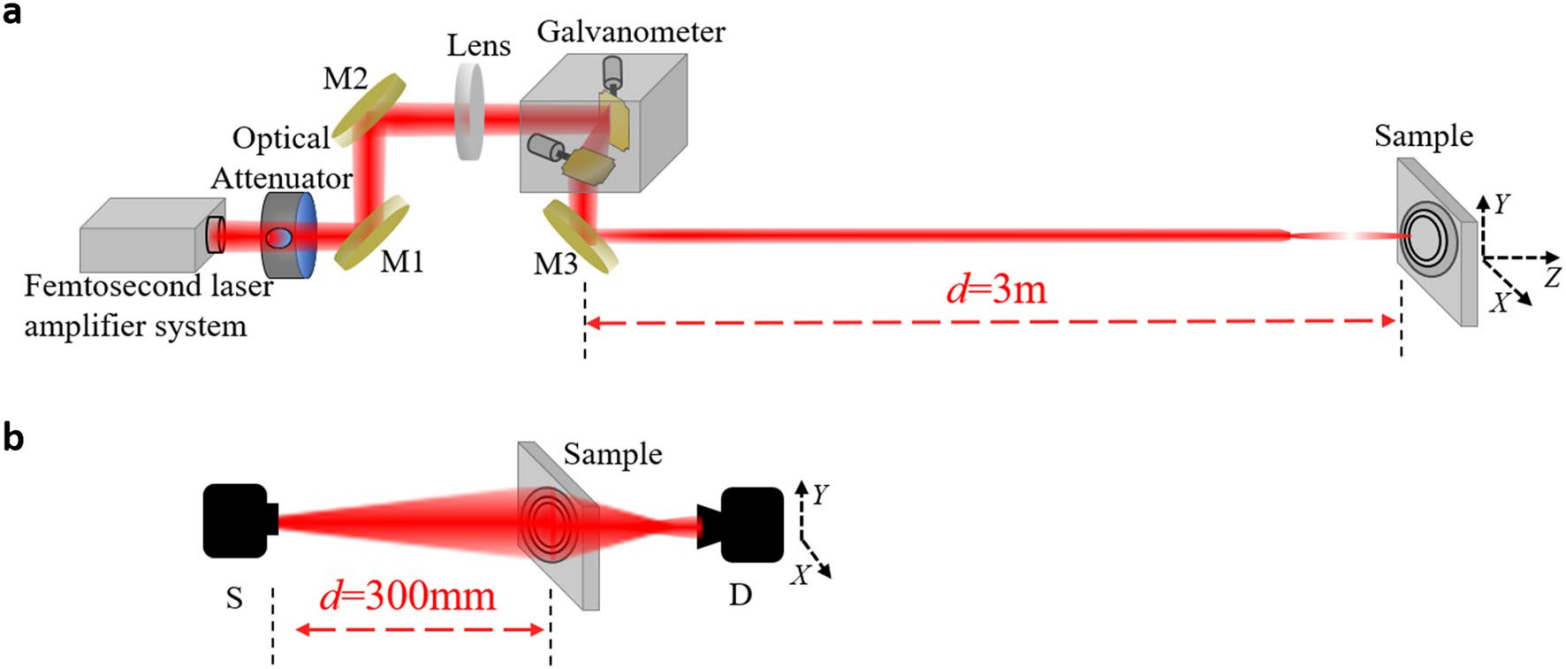
Schematic diagrams of the experimental setup for fabricating (**a**) and characterizing (**b**) SZPs. In (**b**), S represents a 0.1 THz light source (IMPATT diodes, Terasense Inc.) with an output power of 95 mW, and D represents a THz detector (WR-10 ZBD, Virginia Diodes Inc.) with a frequency detecting range of 75–110 GHz, which is mounted on a 2D stepper motor driving translation stage.

The experimental setup is schematically shown in Fig. 1a. A commercial Ti: sapphire femtosecond laser amplifier system (Legend elite, Coherent) produced 800 nm femtosecond laser pulses with a pulse width of 42 fs and a repetition rate of 2.5 kHz. An optical attenuator comprised of a half wave plate and a Glan prism was employed to adjust the laser energy. After reflected by a pair of high reflective mirrors M1 and M2, the pulse train was focused by a lens (*f* = 3,000 mm) and delivered into a computer controlled two-dimensional galvanometer scanner. After reflected by a high reflective mirror M3, the femtosecond laser filament is produced around the geometrical focus of the focusing lens. During the fabrication process, the laser filament is scanned only in the region where the sliver film needs to be removed. The laser pulse energy was fixed at 0.64 mJ in experiments and a 6-cm-long light filament appears near the focus of the focusing lens. The scanning speed of the filament in the *XOY* plane was set to be 200 mm/s during the experiments.

### Characterizing SZPs

The experimental setup for characterizing the SZPs is shown in Fig. 1b. A 0.1 THz source S (IMPATT diodes, Terasense Inc.) is employed to generate Gaussian CW THz waves with a beam waist diameter of ~ 12 mm (FWHM), a divergence angle of 9.3°, and an average power of 95 mW. The distance between the SZPs and the THz source is 300 mm. The transmitted THz beam after the SZPs was detected by a polarization sensitive THz point detector D (WR-10 ZBD, Virginia Diodes Inc.). In order to record the spatial distribution of the THz beam, the point detector is mounted on a 2D stepper motor driving translation stage and scans in the *XOY* plane with a scanning step of 1 mm. During the experiments, the THz detector is firstly rotated when it is centered on the optical axis until the signal value reaches the maximum, making sure that the polarization state of the THz wave is consistent with the polarization sensitive direction of the detector. Then, the zone plate is inserted between the source and the detector.

## Results and discussions

### THz vortex beams obtained by SZPs

Based on the radial Hilbert transform, in the polar coordinates (*r*, *φ*), the phase function of the designed THz SZPs can be expressed as^32^:

1 $$F\left( {r,\varphi } \right) = \exp \left( {jl\varphi - \frac{{j\pi r^{2} }}{\lambda f}} \right)$$

where *l* denotes the topological charges, *λ* is the incident wavelength and *f* represents the focal length of the THz SZPs. The binary transmittance function of the THz SZPs can be written as:

2 $$t\left( {r,\varphi } \right) = \left\{ \begin{gathered} 1,\sin \left( {l\varphi - \frac{{\pi r^{2} }}{\lambda f}} \right) \ge 0 \hfill \\ 0,\sin \left( {l\varphi - \frac{{\pi r^{2} }}{\lambda f}} \right) < 0 \hfill \\ \end{gathered} \right.$$

**Figure 2:**
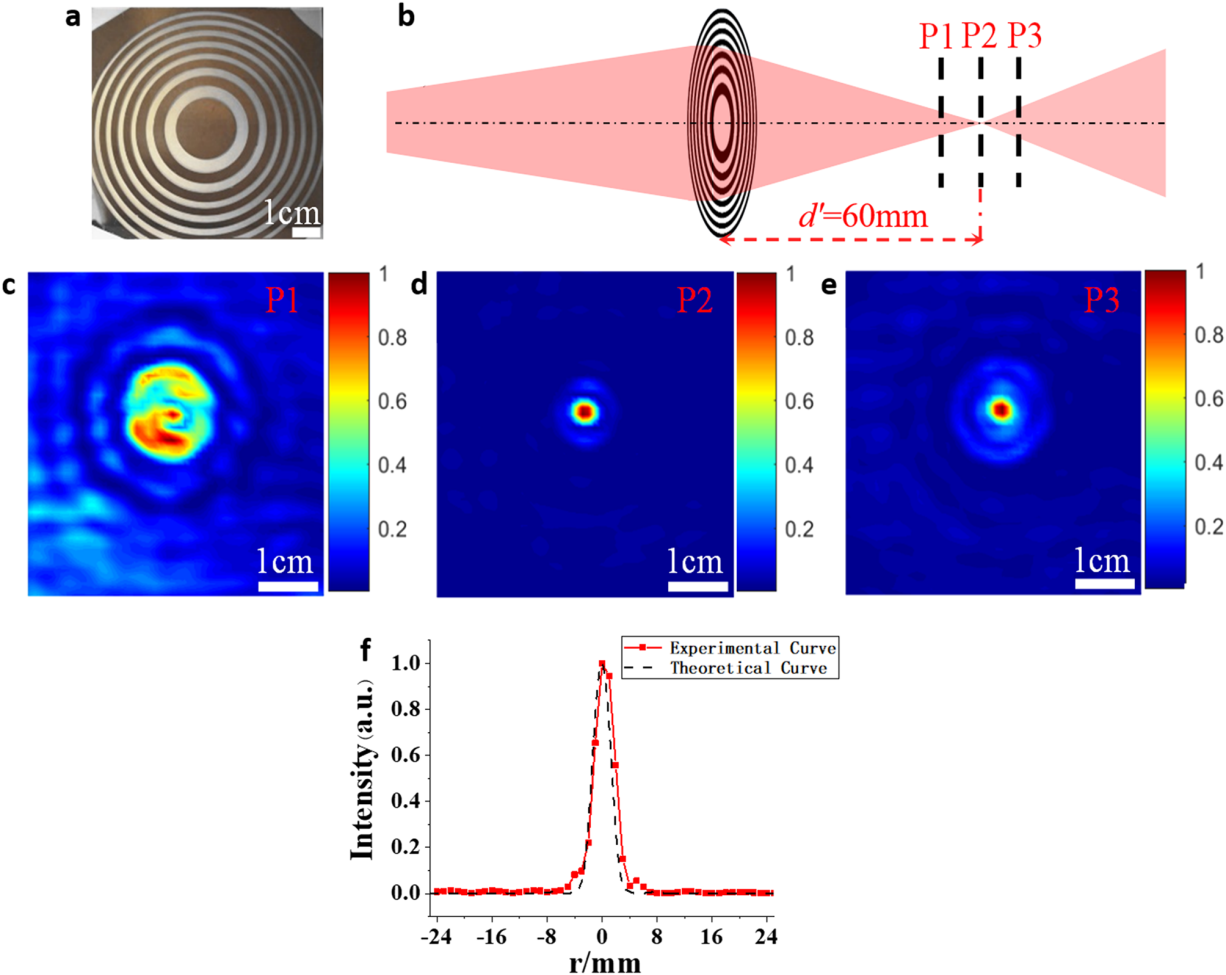
(**a**) Optical image of the SZP with *l* = 0 fabricated by femtosecond laser filament; (**b**) schematic diagram of the measurement locations of the THz intensity distributions near the SZP’s image plane; (**c**–**e**) measured intensity distributions of the THz beam at three different positions (P1–P3); (**f**) experimental and theoretical radial intensity profiles of the THz beam at the SZP’s image plane.

The SZPs’ patterns with different topological charges (*l* = 0, 1, 3) are designed by Eq. (2)^32^. The designed patterns are loaded to the software used to control the galvanometer, and finally the SZPs are fabricated by scanning femtosecond laser filament via the galvanometer. Figure 2a shows the optical image of the fabricated SZP with the topological charge *l* = 0 and a focal length *f* = 50 mm. The performance of this SZP was characterized using the setup in Fig. 1b. According to the Gaussian imaging formula, the image plane of the THz source is 60 mm behind the SZP. The intensity distribution of the THz beam was measured at three different positions in the vicinity of the image plane as is shown in Fig. 2b. The three positions are respectively corresponding to *d’* = 50 mm, 60 mm and 70 mm (see Fig. 2b). The measured intensity distributions are shown in Fig. 2c,e. The measured radial intensity profile at the image plane (P2) is presented as the solid curve in Fig. 2f, which agrees well with the simulation result indicated by the dotted line. The THz beam’s focal spot has a diameter of 3.3 mm (Full width at half maximum, FWHM) at *d’* = 60 mm, which coincides with the design scheme.

**Figure 3:**
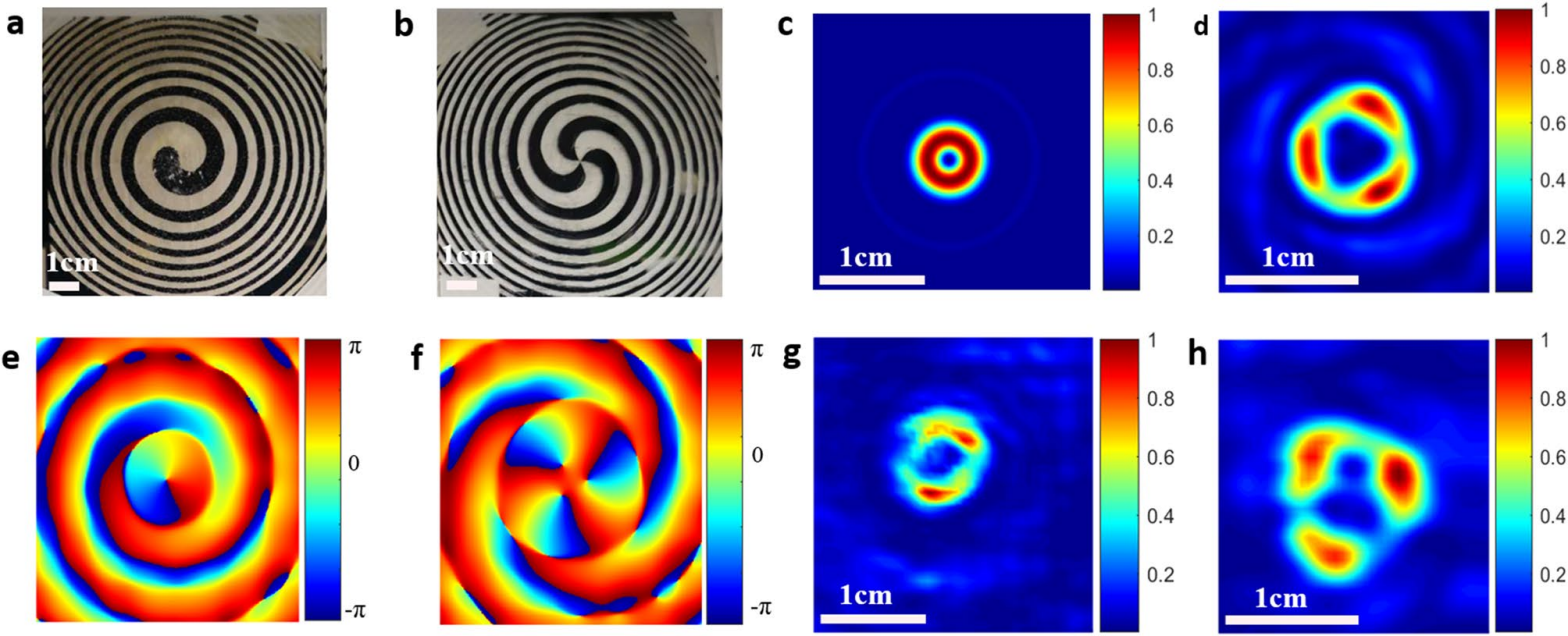
Optical images (**a**,**b**) of the fabricated SZPs with *l* = 1 and 3, respectively; calculated intensity distributions (**c**,**d**) and phase distributions (**e**,**f**) of the THz beams at the image plane of the SZPs with *l* = 1 and 3, respectively; measured intensity distributions (**g**,**h**) of the THz beams at the image plane of the SZPs with *l* = 1 and 3, respectively.

The THz SZPs with topological charges of 1 and 3 were fabricated and the optical images are respectively shown in Fig. 3a,b. Using Kirchhoff diffraction formula^33^, the intensity and phase distribution of THz beam at the image plane of the designed SZPs can be calculated. The calculated results are presented in Fig. 3c,f. The measured THz beam intensity distributions at the image plane of SZPs with *l* = 1 and 3 are respectively shown in Fig. 3g,h, which agree well with the calculation results.

It should be noted that the quartz substrate is not damaged after the removal of the silver film by the laser filament, because the laser fluence at the target surface is calculated to be 2.1 J/cm^2^ (the product of the clamping intensity 5 × 10^13^^31^ and the pulse duration 42 fs) lower than the quartz damage threshold of 3 J/cm^2^^34^. Furthermore, except for the quartz substrate, sapphire and diamond with high THz transmittance are both potential substrates for THz elements, which cannot be damaged by femtosecond laser filament due to their high damage thresholds^35,36^.

### Spatial resolution of the direct writing system

**Figure 4:**
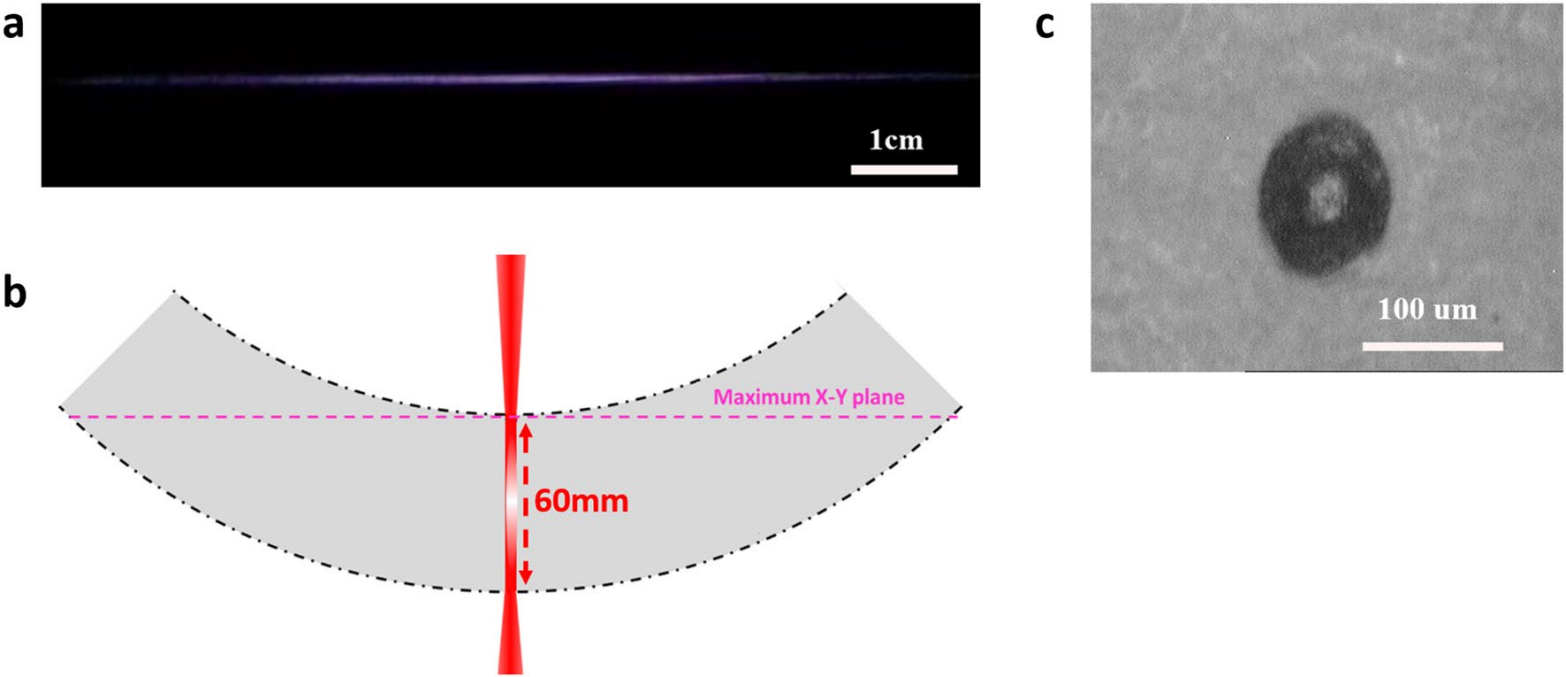
Estimation of the maximal direct writing range. (**a**) Optical image of the laser filament taken by a CCD camera; (**b**) schematic diagram of the laser filament direct writing range (gray area is the area covered by the filament when it is scanned by the galvanometer); (**c**) burn pattern on a thermal paper caused by the laser filament used in the fabrication process.

**Figure 5:**
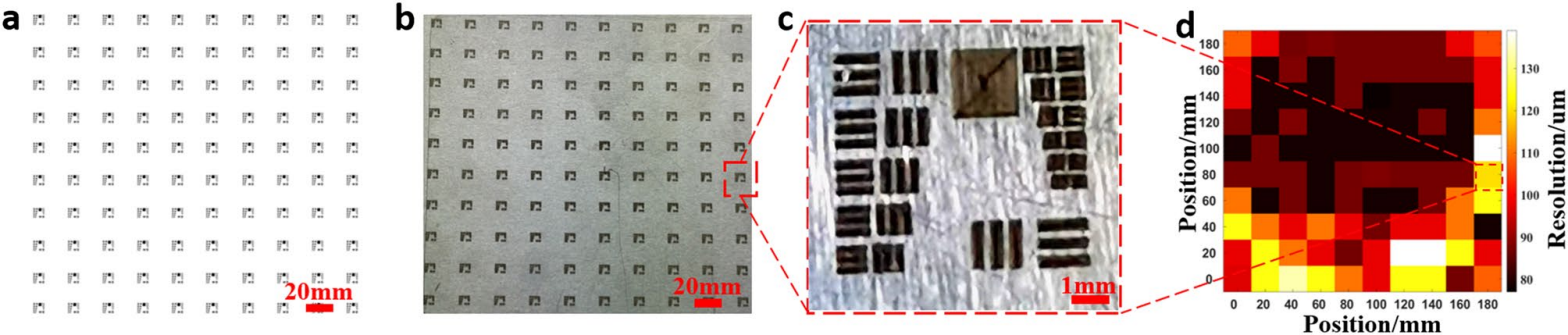
Spatial resolution performance measured by fabricating resolution test chart array on an aluminum foil sample. (**a**) Schematic diagram of the resolution chart array that will be written onto the sample surface; (**b**) photo of the fabricated resolution chart array on the sample; (**c**) enlarged image of the resolution chart fabricated at the edge of the fabrication region; (**d**) distribution of the fabrication resolution in the *XOY* plane; each resolution chart stands for an area of 20 × 20 mm^2^, and its position is one-to-one corresponding to (**b**).

The spatial resolution of the femtosecond laser filament direct writing system determines the performance of the fabricated THz elements, so it should be investigated in detail. Figure 4a shows the optical image of the femtosecond laser filament used in our experiments. The length of the filament is about 6 cm. Although the maximal fabrication range in the *XOY* plane is calculated to be 1.194 × 1.194 m^2^ using the geometries in Fig. 4b and assuming an axially uniform filament, the maximal fabrication range demonstrated experimentally is only 200 × 200 mm^2^ due to the limited size of the mirror M3 in Fig. 1a. To investigate the spatial resolution of the fabrication system, the resolution chart (USAF-1951) array shown in Fig. 5a was directly written onto the surface of an aluminum foil sample (200 × 200 × 0.3 mm^3^) and the fabrication result is shown in Fig. 5b. The zoom-in picture of the fabricated resolution chart in the periphery of the fabrication range is shown in Fig. 5c. It is seen that no defocus induced resolution loss appears in this image. By measuring the minimum feature size that can be achieved in each resolution chart, the spatial distribution of the fabrication resolution on the whole sample surface can be obtained, which is shown in Fig. 5d. The mean resolution and its standard deviation of the direct writing system are 93.9 μm (about λ_THz_/33) and 16.6 μm respectively. The ~ 100 µm resolution is consistent with the diameter of the filament which can be measured by a thermal paper. The thermal paper is inserted perpendicular to the filament and the cross-sectional filament intensity pattern is recorded, which is shown in Fig. 4c. The diameter of the laser filament could be adjusted by controlling the focal length of the focusing lens, the laser peak power and the ambient air pressure. For example, by simultaneously controlling the focal condition and laser peak power, the filament diameter can be adjusted in the range of 30–100 μm^37^. By increasing the air pressure from 1 to 4 atm, the filament diameter can be reduced by 50%^38^. Therefore, the laser filament with a diameter of 15 μm is expected by simultaneously modulating the focal condition, the laser peak power and the air pressure. As a consequence, the fabrication resolution of 15 μm may be achieved and 6G elements working at 0.6 THz with similar performance to those in our experiments may be fabricated by the method proposed in this paper.

## Conclusions

In this paper, we propose a high throughput fabrication method based on the laser induced filamentation for the fast fabrication (~ 4 min) of large-size (100 × 100 mm^2^) THz SZPs with topological charges *l* = 0, 1 and 3. The intensity distribution of the THz beam in the image plane (*d’* = 60 mm) of the fabricated SZPs is measured which agrees well with the designed distributions. The maximal element size fabricated by our system is 200 × 200 mm^2^. Meanwhile, the galvanometer scanner used in our experiments offers a fast fabrication speed (200 mm/s) and the fabrication of one SZP sample takes ~ 4 min.

By directly writing the standard resolution test chart (USAF-1951) array on a large-size aluminum sample, the mean resolution (93.9 μm) and its standard deviation (16.6 μm) of the laser filament direct writing system are determined. It is expected that by changing the laser power and focal condition, the fabrication resolution can be further improved which will be investigated in future researches. It concludes that the fast, high throughput, large scale laser filament direct writing method can find important applications in fabricating the large aperture elements, such as those used in the next generation wireless communications.

## Data Availability

The datasets generated and analyzed during the current study are available from the corresponding author on reasonable request.
